# Nitric oxide is involved in the brassinolide-induced adventitious root development in cucumber

**DOI:** 10.1186/s12870-020-2320-y

**Published:** 2020-03-06

**Authors:** Yutong Li, Yue Wu, Weibiao Liao, Linli Hu, Mohammed Mujitaba Dawuda, Xin Jin, Zhongqi Tang, Jianjun Yang, Jihua Yu

**Affiliations:** 1 College of Horticulture, Gansu Agricultural University, Lanzhou, 730070 People’s Republic of China; 2 Department of Horticulture, FoA, University for Development Studies, P. O. Box TL 1882, Tamale, Ghana

**Keywords:** Brassinolide, Nitric oxide, Nitric oxide synthase, Nitrate reductase, Nitrate reductase genes

## Abstract

**Background:**

Brassinolide (BR), as a new type of plant hormones, is involved in the processes of plant growth and stress response. Previous studies have reported the roles of BR in regulating plant developmental processes and also response tolerance to abiotic stresses in plants. The main purpose of our study was to explore whether nitric oxide (NO) plays a role in the process of BR-induced adventitious root formation in cucumber (*Cucumis sativus* L.).

**Results:**

Exogenous application of 1 μM BR significantly promoted adventitious rooting, while high concentrations of BR (2–8 μM) effectively inhibited adventitious rooting. NO donor (S-nitroso-N-acerylpenicillamine, SNAP) promoted the occurrence of adventitious roots. Simultaneously, BR and SNAP applied together significantly promoted adventitious rooting and the combined effect was superior to the application of BR or SNAP alone. Moreover, NO scavenger (c-PTIO) and inhibitors (L-NAME and Tungstate) inhibited the positive effects of BR on adventitious rooting. BR at 1 μM also increased endogenous NO content, NO synthase (NOS-like) and Nitrate reductase (NR) activities, while BRz (a specific BR biosynthesis inhibitor) decreased these effects. In addition, the relative expression level of *NR* was up-regulated by BR and SNAP, whereas BRz down-regulated it. The application of NO inhibitor (Tungstate) in BR also inhibited the up-regulation of *NR*.

**Conclusion:**

BR promoted the formation of adventitious roots by inducing the production of endogenous NO in cucumber.

## Background

Brassinolide (BR), a new plant hormone, was first discovered during the screening of pollen grains [1]. As a steroid hormone, it plays an important role in regulating various developmental processes, including root and hypocotyl elongation [2] . Moreover, it mediates plant responses to various stimuli, such as hypoxia stress [3], chilling injury [4], salt stress [5], heavy metal stress [6] and drought stress [7]. Yuan et al. [8] reported that the application of 2,4-Epibrassinolide (EBL) alleviated Ca(NO_3_)_2_ stress in cucumber plants by regulating mineral nutrients uptake and distribution. Zhao et al. [9] also found that exogenous EBR application ameliorated the inhibitory effects of photosynthesis, antioxidant enzyme activity and Rubisco activase (RCA) gene expression in *Triticum aestivum* induced by a combination of drought and heat stress. The effect of BR on plant growth and development processes depends on the concentration. Low concentration of BR was suitable for callus growth and shoot regeneration in *Spartina patens* [10], while high concentration of epibrassinolide inhibited the growth of *Brassica oleraceae* cotyledons [11]. Brassinazole (BR_Z_) is a specific BR biosynthesis inhibitor. BR_Z_-treated cress showed dwarfism, with altered leaf morphology, including the downward curling and dark green color typical of *Arabidopsis* BR-deficient mutants and the application of 10 nM brassinolide could reverse the dwarfism [12].

Nitric oxide (NO), a ubiquitous signal molecule, plays important roles in different plant tissues and participates in a variety of physiological processes [13]. Many researchers observed that NO induced root development in *Zea mays* [14], and it also induced seed germination, seedling development, stomatal responses, senescence, flowering and protection against pathogens in different plant species [15–20]. NO production in plants has two pathways, including enzymatic pathway and non-enzymatic pathway. Nitrate reductase (NR) and NO synthase (NOS)-like enzyme are the NO-producing enzymes identified in plants [21]. Zhu et al. [22] have reported that NO production through NOS and NR pathways was involved in adventitious rooting of cucumber explants induced by H_2_. The activities of NR and NOS-like enzymes were involved in BR signaling [23]. Moreover, as the second messenger, NO could interact with some hormones to regulate plant physiological and biochemical responses. It is involved in the signaling pathways of salicylic acid (SA), cytokinin (CTK), jasmonic acid (JA), ethylene (ETH), hydrogen peroxide (H_2_O_2_) and indole-3-acetic acid (IAA) [24–28]. Pagnussat et al. [29] reported the role of IAA and NO in the signaling pathway during the effect of exogenous IAA on the adventitious roots of cucumber. It was clarified that NO operates downstream of IAA promoting adventitious root development through the GC-catalyzed synthesis of cGMP. Both NO and H_2_O_2_ played crucial roles and had synergistic effect on adventitious root development in marigold (*Tagetes erecta* L.) [30].

The formation of adventitious roots is a fundamental process of root biology, through which cells of adventitious roots form new roots after the embryo. The development of adventitious roots is a complex process regulated by various environment and plants hormones factors [31, 32]. Pagnussat et al. [27] observed that a transient increase in NO concentration was required and was part of the molecular events involved in adventitious root development induced by indole acetic acid (IAA), indicating that NO mediates the auxin response leading the adventitious root formation. BR-enhanced water stress tolerance in maize plants was due to BR-induced NO production and NO-activated ABA biosynthesis [33]. The existence of a signaling pathway leading to BR-mediated systemic virus resistance involves local Respiratory Burst Oxidase Homolog B (RBOHB)-dependent H_2_O_2_ production and subsequent systemic NR-dependent NO generation [34]. Kwak et al. [35] reported that lower concentrations of BL increased the number and length of adventitious roots while higher concentrations of BL caused trichome-like roots. As mentioned above, both BR and NO could promote adventitious root development, which suggest a possible relationship between BR and NO. Karpets and Kolupaev [36] reported that NO was involved in 2,4-epibrassinolide-induced heat resistance of wheat coleoptiles and the functional interaction between NO, ROS, and calcium ions as the signal mediators. Until now, many researches focused on studying the relationship between NO and other plant hormones [24–27]. However, little is known about the relationship between BR and NO during the development of adventitious roots. To explore this issue, pharmacological experiments were conducted using cucumber (*Cucumis sativus* L.) as test material to investigate the role of NO in BR-induced adventitious roots development. The results provide new insights into the involvement of NO in BR-induced adventitious roots development in cucumber.

## Result

### BR concentrations affect number and length of adventitious roots

**Fig. 1:**
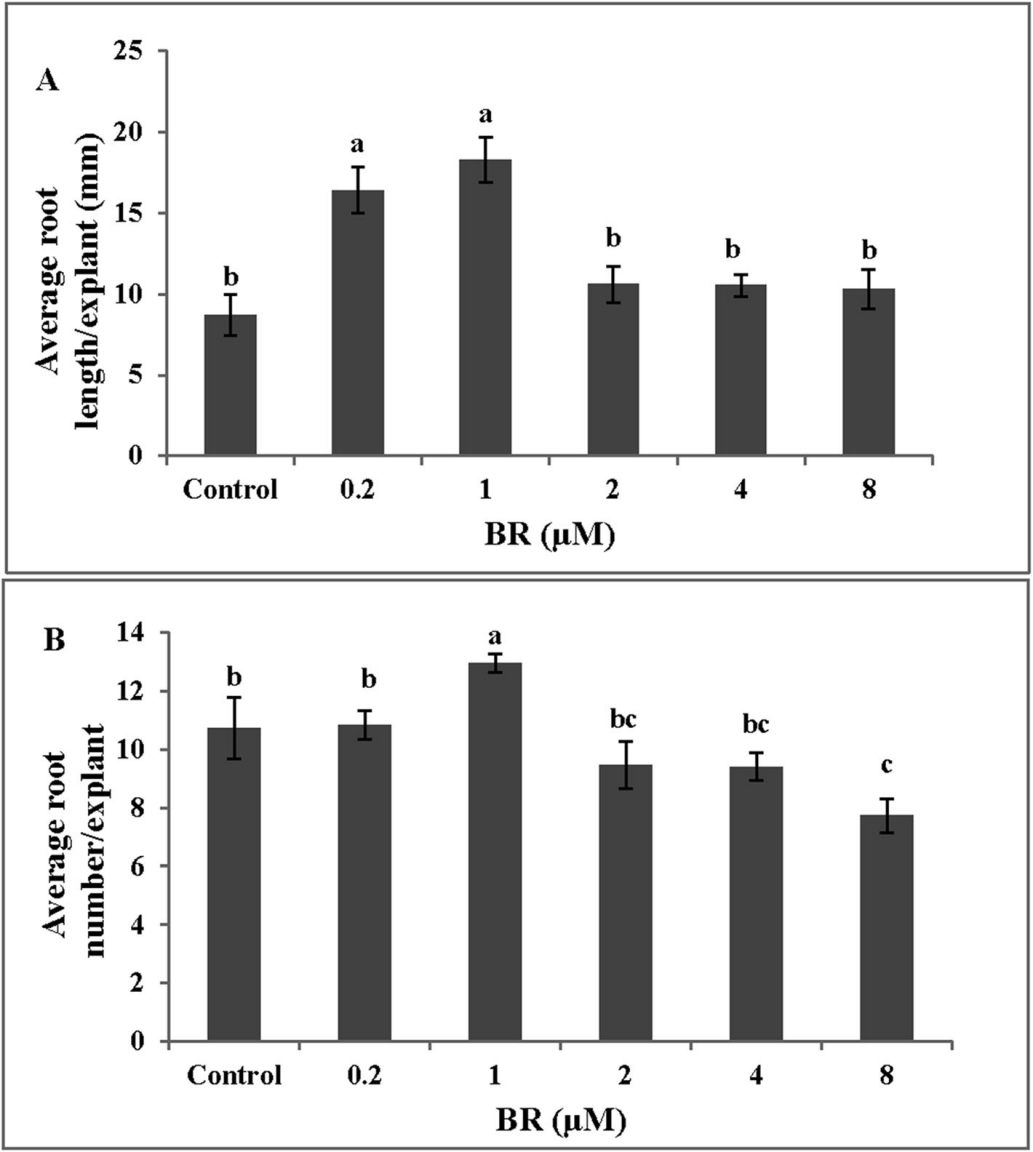
Effect of different concentrations of BR on the average root length and the average root number of cucumber adventitious roots development. Photographs were taken after 5 days of treatment. The different concentration of BR adventitious root length (**a**) and root numbers (**b**) were expressed as means ± SE (*n* = 10 explants from three independent experiments). Bars denoted by different letters were significantly different according to Duncan’s multiple test (*P < 0.05*)

In order to investigate the effect of BR on adventitious roots, the cucumber explants were exposed to different concentrations of BR (0, 0.2, 1, 2, 4 and 8 μ M). The root length and root number of adventitious roots initially increased and then decreased with the increase of BR concentration, both reaching the maximum values at 1 μM (Fig. 1). Thus, the optimum concentration of BR (1 μM) was used in the subsequent experiment.

### Number and length of adventitious root are affected by NO scavenger and inhibitors

**Fig. 2:**
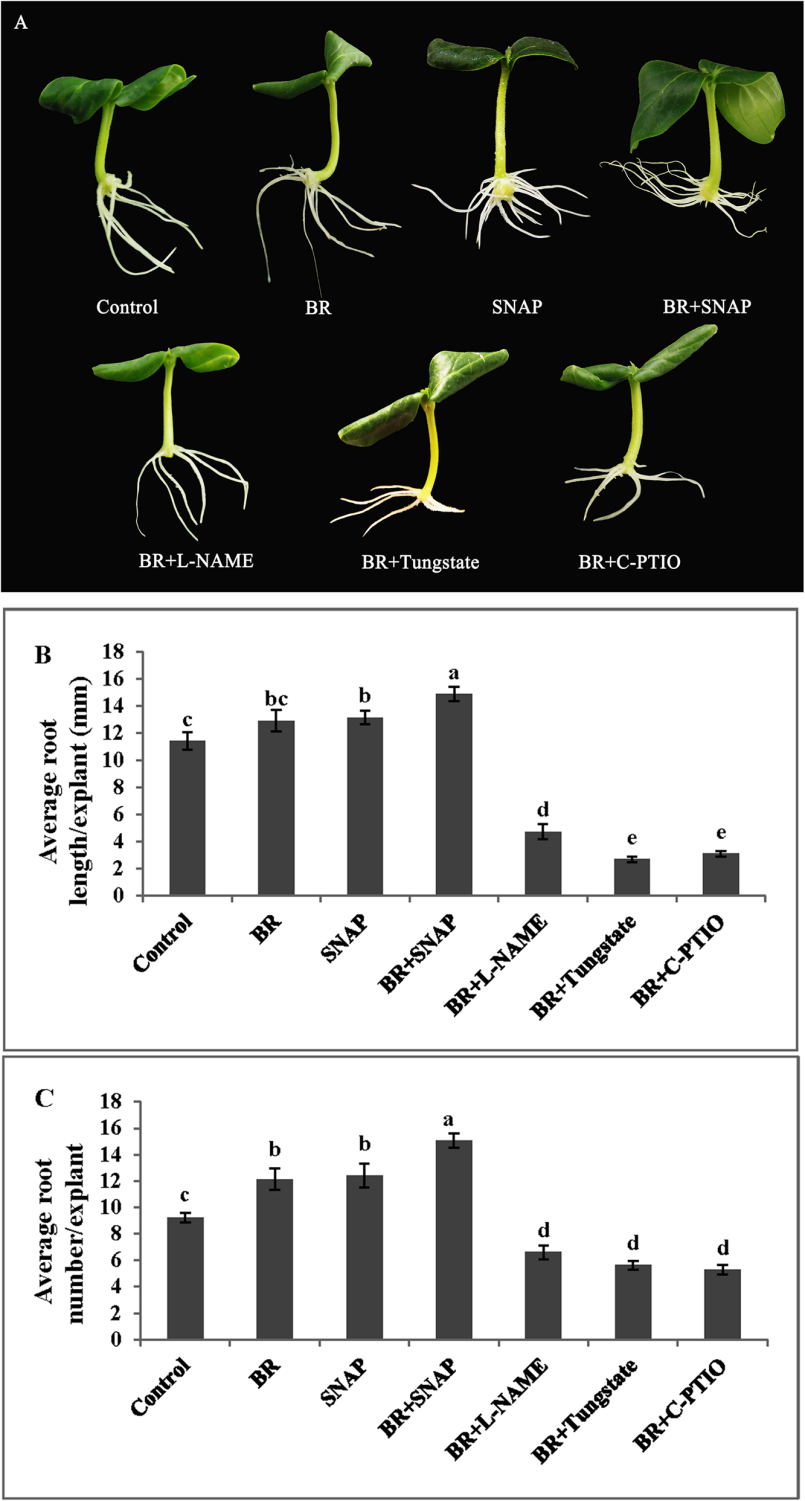
Effect of NO scavenger or inhibitors on BR-induced adventitious root formation. The primary root was removed from hypocotyls of 5-day-old germinated cucumber seedling. Explants were incubated in distilled water, 1 μM BR, 50 μM SNAP, 20 μM L-NAME, 100 μM Tungstate, and 200 μM c-PTIO for 5 days. Photographs were taken 5 days after treatment (**a**). Adventitious root length (**b**) and root numbers (**c**) were expressed as means ± SE (*n* = 10 explants from three independent experiment). Bars denoted by different letters were significantly different according to Duncan’s multiple test (*P* < 0.05)

The effect of NO scavenger (c-PTIO), NOS-like enzyme inhibitor (L-NAME) and NR inhibitor (Tungstate) on BR-induced adventitious rooting was investigated. As shown in Fig. 2, compared with BR treatment, 200 μM c-PTIO, 20 μM L-NAME or 100 μM Tungstate applied in combination with BR treatment significantly inhibited adventitious root formation. The adventitious root number and length of explants treated with SNAP (NO donor) plus BR were significantly higher than those of explants treated with either BR or SNAP alone.

### Temporal regulation of endogenous NO content, NOS-like and NR activity by BR

**Fig. 3:**
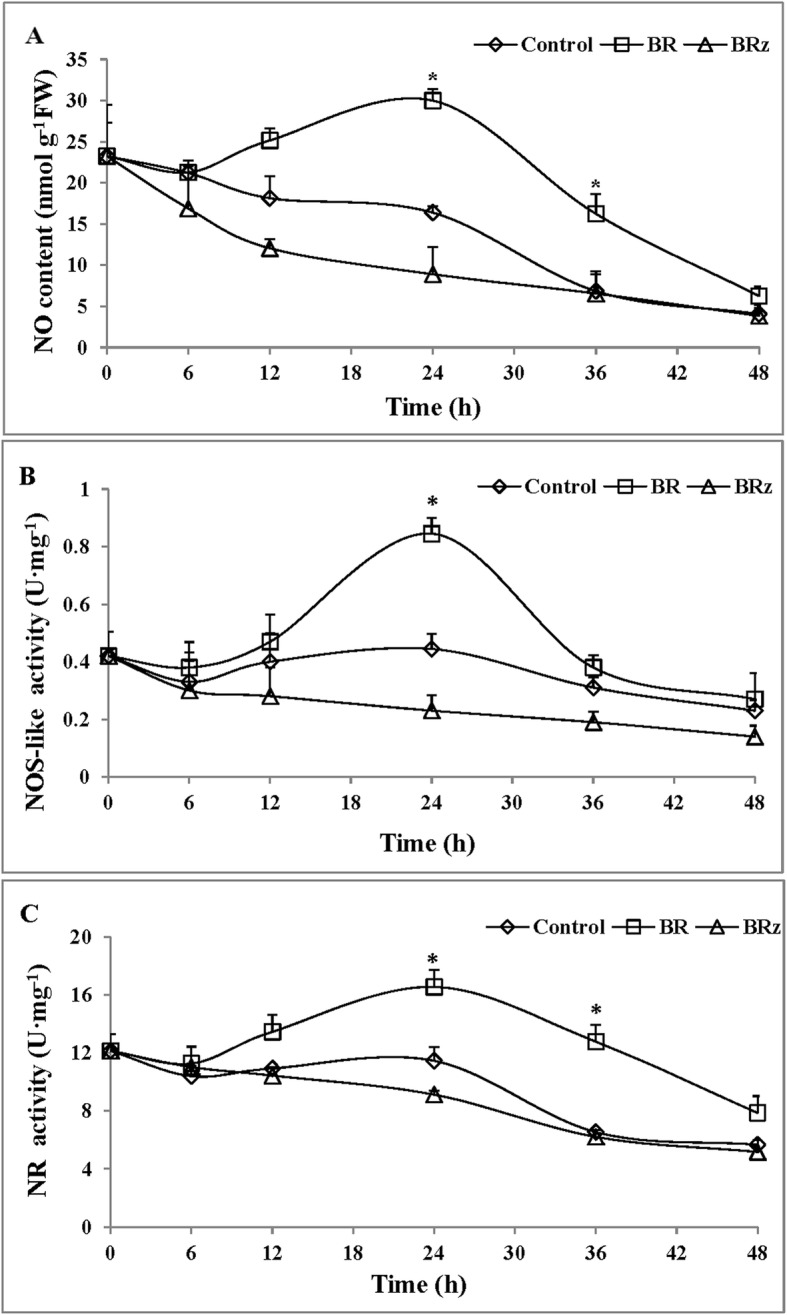
Effect of BR on endogenous NO content, NOS-like and NR activities in a time- dependent manner. The primary root system was removed from the hypocotyls of 5-d-old germinated cucumber seedlings. Enzymatic activities were measured from 1 cm-long segment taken from the base of hypocotyl. NO levels (**a**) were determined by Griess reaction reagent in hypocotyls of explants treated with distilled water (control) or 1 μM BR, 1.5 μM BRz. NOS-like (**b**) and NR activities (**c**) of hypocotyls (1 cm) were determined using the NOS-like and NR determination Kit (Jiancheng, Nanjing, China) according to the manufacturer’s instructions in explants treated with distilled water (control) or 1 μM BR,1.5 μM BRz. Values (means ± SE) are the averages of three independent experiments (*n* = 15 explants from each of three independent experiments). Asterisks indicate that mean values are significantly different between treatments and control (*P* < 0.05) according to Duncan’s multiple test

The time-course of NO content as affected by 1 μM BR or 1.5 μM BR_Z_ treatment is shown in Fig. 3a. Compared with the control, the NO content of BR-treated explants has a slow downward trend at 0 h–6 h, which may be due to the wound response. From 6 h to 24 h, the NO content of BR-treated explants increased, and subsequently gradually decreased until 48 h (Fig. 3a). The content of NO in the BR treatment reached the maximum at 24 h and was about 1.8 times as compared to the control. In addition, the NO content of BRz-treated explants gradually decreased from 0 h to 48 h, and the NO levels was always lower than those of BR-treated explant. Thus, the data suggest that BR regulated endogenous NO to promote the development of adventitious roots in cucumber.

To explore the relationship between BR and NO, we further analyzed the effects of BR on the activities of NOS-like and NR enzymes in cucumber explants during the formation of adventitious roots (Fig. 3b and c). The application of BR distinctly affected the time course of NOS-like activity. The NOS-like activity in BR-treated explants decreased slightly at 0–6 h, and then increased from 6 h to 24 h, reaching a maximum at 24 h, which was about 2-fold of the control. Then, from 24 h to 48 h, NOS-like activity tended to gradually decreased (Fig. 3b). Meanwhile, compared with the control and the BR treatment, the NOS-like enzyme activity of BR_Z_ treatment consistently decreased throughout the whole experiment (Fig. 3b). Similarly, the NR activity of explants treated with BR decreased transiently during the first 6 h period, followed by a significant increase from 6 h to 24 h, which reached its highest activities at 24 h (about 1.4-fold of the control), and then decreased at 48 h (Fig. 3c). However, the NR activity in BRz-treated explants continuously decreased during the time of experiment (Fig. 3c). In conclusion, the activity of NOS-like and NR enzymes were promoted by BR treatment, while the BRz inhibited the activities of these two enzymes. Here, we showed that BR regulated the production of endogenous NO by inducing the increase activity of NOS-like and NR enzyme during the adventitious root formation.

### NO content, NOS-like and NR activity under BR, SNAP, L-NAME, tungstate and BR_Z_ treatments

**Fig. 4:**
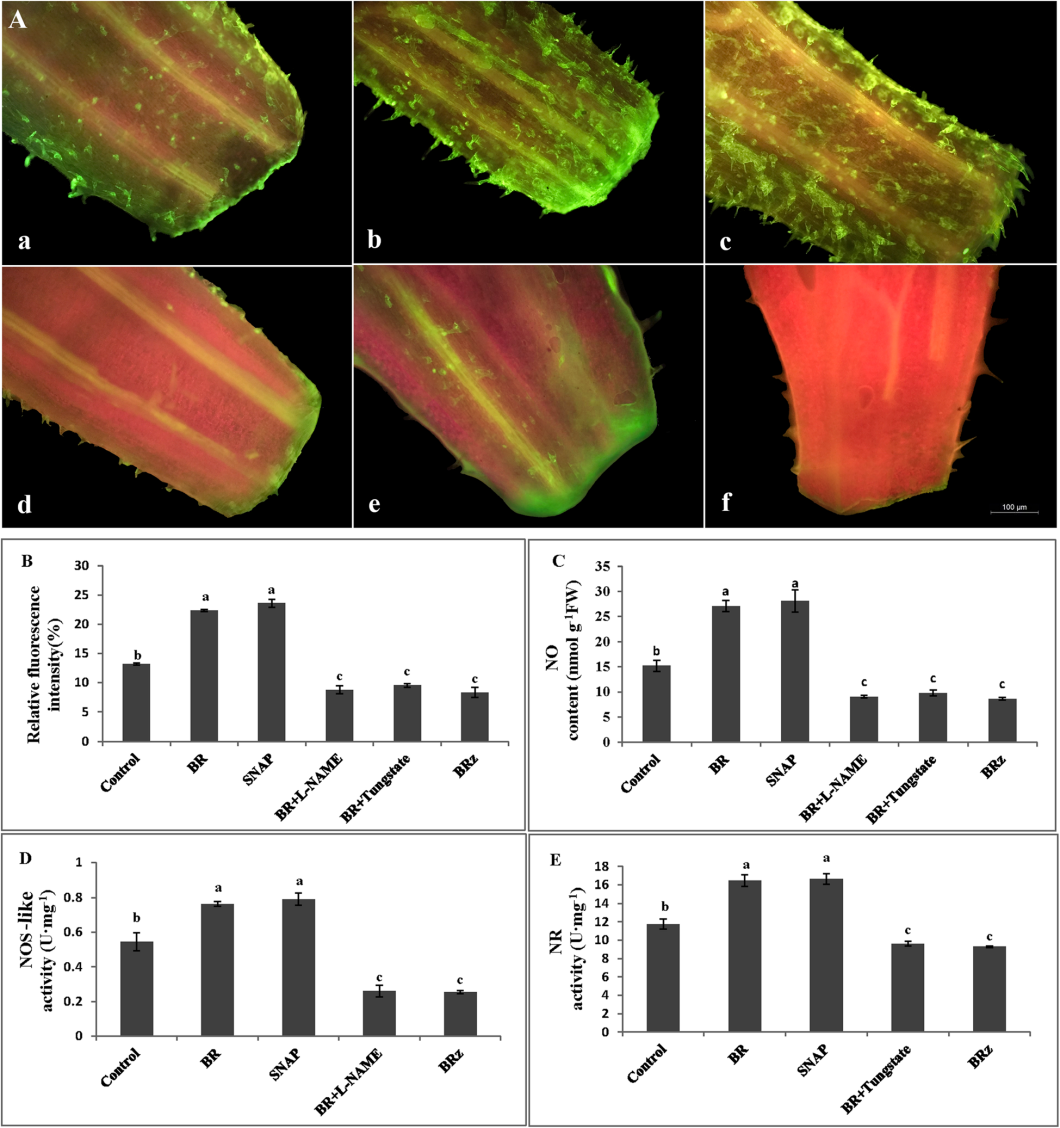
Effects of BR, SNAP, L-NAME, Tungstate and BRz on the content of NO and BR-induced NOS-like and NR activities. The fluorescence localization of NO in hypocotyl of cucumber explants (**A**) treated with control (distilled water) (**a**), 1 μM BR(**b**), 50 μM SNAP (**c**), BR + 20 μM L-NAME (**d**) and BR + 100 μM Tungstate (**e**), 1.5 μM BRz (f) for 24 h. Fluorescence intensity was analyzed using Image J software and expressed as a percentage of the control (**B**). The quantification of endogenous NO content was done in hypocotyl of cucumber explants (**C**). NOS-like (**D**) and NR (**E**) enzymatic activities were measured from 1 cm-long segment taken from the base of hypocotyl. Mean and SE values were calculated from three independent experiments (*n* = 15). Bars denoted by the same letter did not differ significantly at P < 0.05 according to Duncan’s multiple test

In order to further verify whether NO participates in BR-induced adventitious roots formation in cucumber, the explants were placed in BR, SNAP, BR + L-NAME, BR + Tungstate and BRz treatment for 24 h. The fluorescence localization of NO in hypocotyl, the content of endogenous NO and the activities of NR and NOS-like enzymes were analyzed. As shown in Fig. 4a and b, after treatment with BR and SNAP, brighter green fluorescence was observed in the tissue at the place where hypocotyl produce adventitious roots, and the intensity of fluorescence was significantly higher than in control explants, indicating that the production of NO was sharply rising. In opposite, explants treated with BR+ L-NAME, BR+ Tungstate and BRz showed a lower fluorescence in the hypocotyl than in the control plants (Fig. 4a and b). In order to support the qualitative analysis, the quantification of endogenous NO content was done in hypocotyl of cucumber explants. As shown in Figs. 4c, compared with the control, endogenous NO content after treatment with BR and SNAP was significantly increased by 78.03 and 84.79%, respectively. Compared with the BR treatment, when L-NAME and Tungstate were added to the BR solution, the effects of BR were reversed. Indeed, NO content was reduced by 66.5 and 63.8%, respectively (Fig. 4c). Moreover, BR_Z_ treatment alone significantly reduced NO content by 68.1% compared with the BR treatment (Fig. 4c). The qualitative and quantitative analyses of NO in hypocotyl of cucumber explants showed that exogenous application of BR and SNAP significantly increased the production and distribution of endogenous NO in cucumber hypocotyl. As shown in Fig. 4d and e, BR-induced NOS-like and NR activity were blocked by L-NAME and Tungstate. Compared with the control, application of BR and SNAP alone significantly increased the activity of NOS-like enzyme by 40.24 and 45.22%, respectively (Fig. 4d). Moreover, BR + L-NAME and BRz treatments markedly reduced NOS-like enzyme activity by 65.92 and 66.97% compared with the BR treatment, respectively (Fig. 4d). Similarly, compared with the control, the activity of NR after BR and SNAP treatment was significantly increased by 40.17 and 41.53%, respectively (Fig. 4e). Compared with the BR treatment, the activity of NR enzyme after BR + Tungstate and BR_Z_ treatment was drastically reduced by 41.65 and 43.59%, respectively (Fig. 4e). Thus, BR induced the generation of NO by regulating the activity of NOS-like and NR enzymes, and promoted adventitious root formation in cucumber explants.

### The relative expression of *NR* gene under BR, BR_Z_, SNAP, and tungstate treatments

**Fig. 5:**
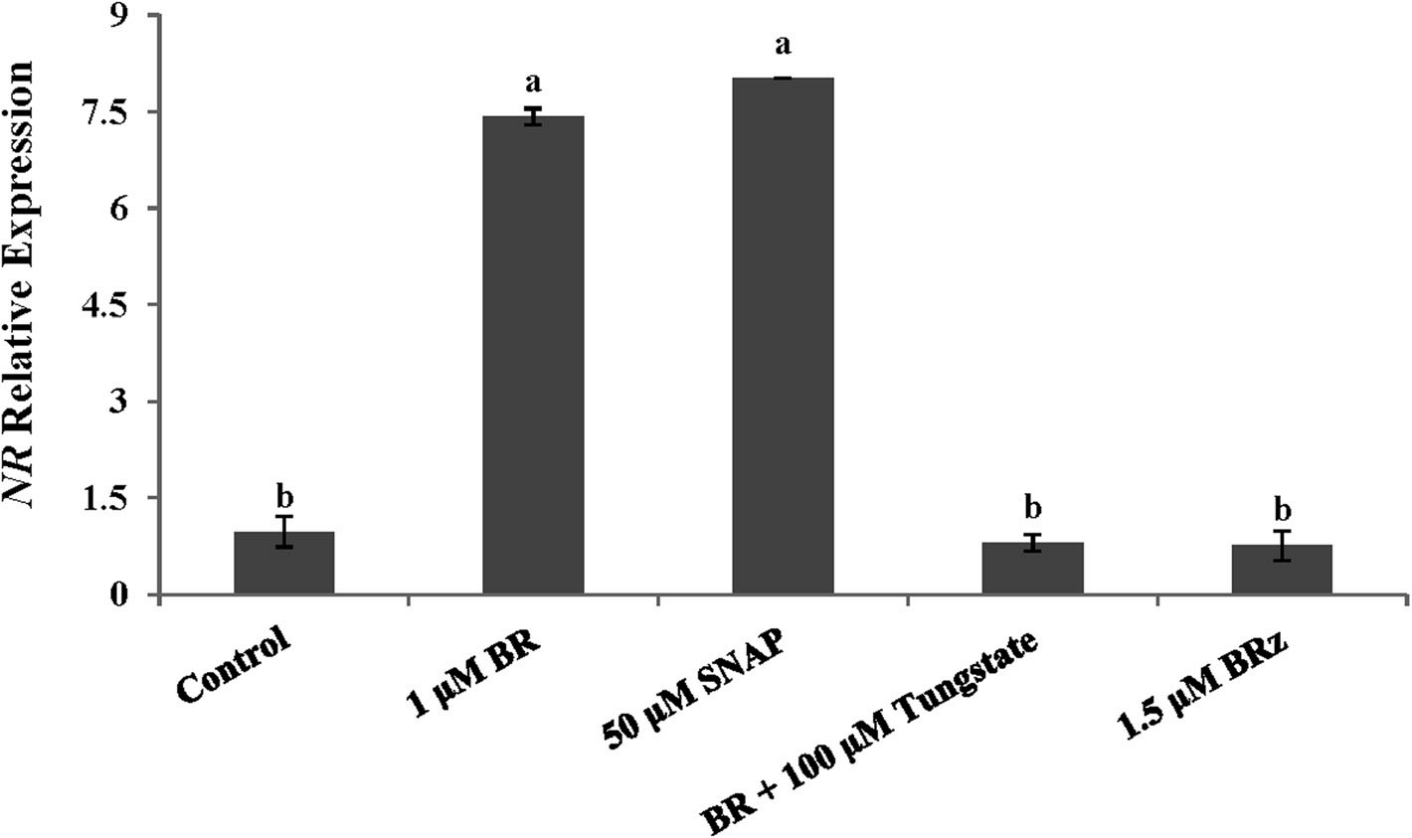
Analysis by qRT-PCR of the effect of BR, SNAP, tungstate and BRz on the expression of *NR* gene in cucumber explants. Explants were incubated for 24 h in water (control) or in the presence of the different compounds in concentration as indicated on the figure. Relative expression was determined using the ΔΔCt method [37], taking *actin* gene as a reference. Data were collected from 3 independent biological replicates. Each replicate represents a pool of ten explants. Bars denoted by identical letters did not differ significantly (Duncan’s multiple test; *P* < 0.05)

During the adventitious rooting process, we performed real time RT-PCR to measure the relative expression of *NR* gene (Fig. 5). Compared with the control, the *NR* expression levels in BR- and SNAP- treatment were significantly higher than those in the control at 24 h after treatment, which were 642.3 and 701.2% higher over in the control (Fig. 5). There was no significant difference in the relative expression level of *NR* between BR + tungstate and the control. The relative expression level of *NR* gene decreased by 89.15 and 89.69% in BR + Tungstate and BR_Z_ treated explants compared with the BR treatment, respectively (Fig. 5).

## Discussion

The data reported here demonstrated the interaction between BR and NO during adventitious rooting in cucumber explants. The results showed that exogenous BR enhanced the number and length of adventitious root at low concentrations, while high concentrations of BR treatments suppressed adventitious root development (Fig. 1), which suggested that the effect of BR on adventitious root is a dose-dependent response. Previous studies showed that the application of BR (0.1–10 μM) promoted hypocotyl elongation in *Arabidopsis* and the influence was related to the concentrations [2, 38]. Similarly, in tomato, Guan and Roddick [39] reported that the application of 24-epibrassinolide (24-EBR) in excess of 1.0 μM reduced root growth as well as root number and root length, but lower concentration of 0.1 μM EBR increased the number and root length. It may be noted that the optimum concentration of BR for rooting in previous research [2, 38] was different from our experiment. It might be due to the sensitivity to BR of the different plant species.

BR has biological activity in the bioassay for auxin, which is similar to the function of gibberellin, ethylene and cytokinin, and it affects root formation and development in plants [40, 41]. BR also participates in the plant life processes, such as responses to various biotic and abiotic stresses [42–44]. Our results also showed that BR promoted adventitious root formation. Furthermore, the exogenous application of BR_Z_ played a vital role in the inhibition of adventitious root development. The results are consistent with that of Kurepin and co-authors who also found that the application of BR_Z_ suppressed hypocotyl growth in *Arabidopsis* and the effect depended on the concentration applied [2]. In addition, the interaction between BR and other hormones also has a certain effect on the growth and development of plants [45–47]. For example, in *Arabidopsis thaliana*, BR and auxin signal transduction have interaction points, which induced the synthesis of auxin [48]. The interaction of Brassinolide and ethylene can control the negative gravitropism of *Arabidopsis* seedlings, and it depends on the signal component of auxin [49]. Compared with a single epibrassinolide (BL) treatment, the foliar application of methyl jasmonate (MeJA) and BL in rice resulted in a remarkable reduction in infection of rice black-streaked dwarf virus (RBSDV) [50]. Moreover, some biological gaseous molecules such as carbon monoxide (CO), NO and hydrogen peroxide (H_2_O_2_) were shown to exhibit similar hormone-like effects as signal transmitters during adventitious rooting [51–53]. It has been confirmed that NO is involved in auxin induced adventitious root formation in marigold [30]. The interaction of NO with other hormones has also been extensively reported [22, 54, 55]. However, the interaction between BR and NO in adventitious root development has hardly been reported. Our results have demonstrated that BR- induced adventitious roots development was inhibited by NO scavenger (c-PTIO) or inhibitors (L-NAME, Tungstate) (Fig. 2). The scavenger and inhibitors of NO not only inhibited the production of endogenous NO, but also inhibited the effect of the exogenous BR. This observation was consistent with the findings of Li et al. [56]. Our results suggested that nitric oxide was involved in the brassinolide-induced adventitious root development in cucumber explants.

In the subsequent experiment, the involvement of endogenous NO in BR-induced adventitious rooting was further confirmed by the increased activities of NOS-like and NR (Fig. 3). BR_Z_ reduced the production of NO and activities of NOS-like and NR, and its effect was time-dependent. Previous studies also showed that methane (CH_4_) triggered the accumulation of NO in cucumber during adventitious root formation [57]. Ethephon treatments induced the increase of endogenous NO level and significantly improved activities of NOS-like and NR during adventitious rooting [58]. Hydrogen gas induced adventitious rooting by enhancing NO level [22]. Hemin might act to promote NO accumulation in mediating adventitious root development of cucumber explants [59]. As indicated above, the endogenous accumulation of NO induced by certain exogenous substance could promote adventitious root development. Therefore, our experiment explained the fact that BR promotes the formation of adventitious root by increasing endogenous NO level and improving the activities of NOS-like and NR enzymes. Recently, it was reported that endogenous NO production and activity of NOS-like and NR were inhibited by c-PTIO in the euhalophyte *Suaeda salsa* [60]. The NO content in tea leaves induced by BR was suppressed when using a NO scavenger [54]. This latest observation was consistent with our results (Fig. 4). We found that exogenous application of BR and SNAP significantly increased endogenous NO production. However, L-NAME and Tungstate, BR_Z_ reversed the positive roles of BR and SNAP in NO accumulation. The induction of NOS-like and NR activities induced by BR were inhibited by L-NAME, Tungstate and BR_Z_. In summary, BR induced the production of endogenous NO by stimulating the activity of NOS-like and NR enzymes and promoted in the development of adventitious roots in cucumber. In the present study, the endogenous NO production and distribution were visualized in planta by the DAF-FM DA fluorescent probe technology, thus verifying that BR could promote the production and accumulation of endogenous NO during adventitious rooting in cucumber. Moreover, NO inhibitors could reverse the role of BR in the promotion of endogenous NO, indicating that NO participated in the process of BR induced adventitious roots in cucumber. Similar studies reported that the production and distribution of endogenous NO were also detected by DAF-2DA, a fluorescent NO indicator dye in *Arabidopsis thaliana* and in maize [33, 61]. Indeed, it is known that NR activity on one hand is regulated by phosphorylation and interaction with 14–3-3 proteins [62, 63], on the other hand, the activity of NR is affected by the abundance of NR enzyme in a certain degree. Whereas the abundance of the NR protein is affected by the accumulation of the *NR* gene transcript. Therefore, we further investigated the regulation of *NR* gene expression. Our data showed that both BR and SNAP induced the accumulation of the transcript, whereas BR_Z_ and Tungstate treatment down-regulated the relative expression level of *NR* (Fig. 5). These results are similar to that of Zhu et al. [22], who showed that 50% hydrogen-rich water (HRW) treatment induced adventitious root development and up-regulated the expression of *NR* in cucumber. Xu et al. [54] also reported that ETH enhanced the relative expression of *NR* and improved adventitious rooting in cucumber. Taken altogether, the data strongly suggest that BR and SNAP stimulate the activity of NR enzyme, inducing the accumulation of transcripts and probably the corresponding protein, causing the production and accumulation of NO and promoting, subsequently, adventitious rooting in cucumber. Similarly, in cucumber, Zhu et al. [22] have reported that nitric oxide is required for hydrogen gas-induced adventitious root formation and Pagnussat et al. [29] have reported that nitric oxide and cyclic GMP are involved in the indole acetic acid-induced adventitious rooting process, which suggests that hydrogen gas and indole acetic acid might be the upstream signal molecule of nitric oxide during adventitious rooting process. It will provide us a new research topic about the up-and-downstream relationship of BR, hydrogen gas and indole acetic acid on adventitious root formation.

## Conclusions

In the present study, we demonstrated that both BR and NO played important role during adventitious roots formation in cucumber explants. BR induced adventitious roots formation by up-regulating the relative expression level of *NR* gene and increasing the activities of NOS-like and NR enzymes, and then improving the endogenous NO level of cucumber explants. In this regard, we argue that NO is involved in BR-induced adventitious root formation in cucumber explant. In our future research, the interaction mechanisms and signal transduction pathway of BR and NO will be investigated by molecular and genetics methods.

## Methods

### Plant material and growth conditions

The seeds of cucumber, *Cucumis sativus* L. var. Xinchun 4, were purchased from Gansu Academy of Agricultural Sciences (Lanzhou, China). The seeds were surface-sterilized in 5% sodium hypochlorite for 10 min, washed with water, germinated in petri dishes with double- layer filter paper moistened with distilled water. The seeds were put in an electronic growth chamber at 25 ± 1 °C for 5 days with a 14-h photoperiod (photosynthetically active radiation = 200 μmol m^− 2^ s^− 1^). Primary roots of 5-day-old seedlings were excised and then the cucumber explants were maintained under the same conditions of temperature and photoperiod for 5 days under different treatments as indicated below. The analytical grade chemicals used in the study were obtained from Chinese companies. Root number and root length per explant were recorded and analyzed.

### Treatments and experimental design

Cucumber explants were placed in petri dishes (diameter = 9 cm) lined with double layer tissue paper and moistened with 60 mL distilled water as control (control) or 60 mL of various concentration (0.2, 1, 2, 4, 8 μM) of Brassinolide (BR, Sigma, USA) and kept at 25 ± 1 °C. The following chemicals were added with suitable concentration of BR: 50 μM SNAP (S-nitroso-N-acerylpenicillamine, Sigma, United Stated), 200 μM c-PTIO (2-(4-carboxy-2-phenyl)-4,4,5,5-tetramethy limidazoline-1-oxyl- 3-oxide, Sigma, United Stated), 20 μM L-NAME (N-nitro-l-arginine methyl ester, Sigma, United Stated) and 100 μM Tungstate (Zhongtai Chemical Co.Ltd. Shanghai, China). Moreover, 1.5 μM BR_Z_ (brassinazole, Sigma, United Stated) was administered to explants. The concentrations of these chemicals were selected based on the results of preliminary experiments (data not shown). The treatments were arranged in a completely randomized design in three replicates. Each experimental unit consisted of ten individual explants from which data were taken. Data are expressed as means ± standard error (SE).

### Determination of root number and length of cucumber explants

Five days after treatments, the root number of each explant was counted and the root length of each explant was measured with a ruler (accuracy is 0.1 cm). Three independent biological replications were done and ten explants of each replication were analyzed. Data were expressed as the average ± SE.

### Determination of the endogenous NO content

The endogenous NO level was measured as described by Liao et al. [30] using the Griess reagent method. Half of gram (0.5 g) of hypocotyls (segments 1 cm long from the base of the hypocotyl) were frozen in liquid nitrogen, then ground with mortar and pestle, and homogenized in 3 mL of 50 mM ice-cold acetic acid buffer (pH 3.6), containing 4% (w/v) zinc diacetate. The homogenates were centrifuged at 10,000×*g* for 15 min at 4 °C and the supernatants were collected.

The pellets were washed using 1.0 mL of the above extraction buffer and centrifuged. Activated charcoal (0.1 g) was added to the supernatant, which was then filtered and the absorbance was determined at 540 nm. The NO content was calculated by comparison with a standard curve of NaNO_2_. Measurements were done in 3 independent biological replicates and data represent the average ± SE.

### NOS-like and NR activity determination

The activities of NR and NOS-like were analyzed by nitrate reductase assay kit and nitric oxide synthase assay kit according to the manufacturer’s instructions. The kits were purchased from Nanjing Jiancheng Biological Engineering Co, China. Measurements were done in 3 independent biological replicates and data represent the average ± SE.

### Imaging of endogenous NO by fluorescence microscope

To follow NO accumulation in planta, hypocotyls (1 cm) of cucumber explants grown on plates were incubated in the dark for 2 h in the presence of 20 μM DAF-FM DA (4-amino-5-methy- lamino-2, 7-diamino-fluorescein diacetate, sigma) prepared in 50 mM Tris-HCl (pH 7.5). Hypocotyls were extensively washed with distilled water to remove excess of the fluorophore. The hypocotyls under each condition were observed with a fluorescent microscope (Leica 400x, Planapo, German Weizla). Fluorescence intensity was analyzed using ImageJ software and expressed as a percentage of the control. The experiment was repeated three times and ten hypocotyls were observed each time.

### Determination of transcript abundance by real-time PCR

Total RNA was isolated from 100 mg (fresh-weight) of excised cucumber hypocotyls (segments 1 cm long from the base of the hypocotyl), ground with mortar and pestle in liquid nitrogen, using plant RNA extraction kit (TaKaRa MiniBEST 9769; TaKaRa Biomedicals, Japan) according to the manufacturer’s instructions. Synthesis of cDNA was performed with Prime Script™ RT reagent Kit (TaKaRa Biomedicals, Japan) starting from 500 ng of total RNA according manual’s instructions. The real time quantitative RT-PCR was used to analyze the relative expression of *NR* genes in hypocotyl of cucumber explant through a SYBR Premix Ex Taq II (Tli RNaseH Plus; TaKaRa Biomedicals, Japan). *Actin* gene (accession number: DQ641117) was used as an internal control. Gene-specific primers were designed by Primer3plus as followed: for *NR* (accession number: JQ692875.1), forward 5′ -AAACCCTACATCCTTCACTCTCG − 3′ and reverse 5′ -GGTCCATTGCCATTTCTCTTCT- 3′, for *actin*, forward 5′-CCCATCTATGAGGGTTACGCC-3′ and reverse 5′- TGAGAGCATCAGTAAGGTCACGA-3′. The total volume of each reaction was 20 μ L, and contained 10 μL SYBR Premix Ex Taq II, 2 μL of 10-fold diluted cDNA and 0.8 μL of 10 μM forward and reserve primers, to a final volume of 20 μL by adding water. Amplification program consisted of one cycle of 95 °C for 60 s, 40 cycles of 95 °C for 5 s, and melting analysis at 60 °C for 20 s, and 95 °C for 15 s, followed by one cycle of 60 °C for 60 s, and 95 °C for 15 s. All qRT-PCR for each gene was performed in three biological replicates, with three technical repeats per experiment. The relative quantification of mRNA levels is based on the method of Livak and Schmittgen [37]. The threshold cycle value (Ct) of *actin* was subtracted from that of the target gene to obtain a ΔCt value. The Ct value of the control sample in experiment was subtracted from the ΔCt value to obtain a ΔΔCt value. The expression level relative to the control for each sample was expressed as 2^-ΔΔCt^.

### Statistical analysis

Each experiment was repeated three times and the data collected were expressed as mean values ± standard error (SE). The analysis of variance was performed using SPSS Statistics 17.0 software and treatment means were separated by Duncan’s multiple range test (*P* < 0.05).

## Data Availability

The datasets generated during the current study are available from the first author on reasonable request.

## Abbreviations

24-EBR: 24-epibrassinolide
BL: epibrassinolide
BR: Brassinolide
BR_Z_: Brassinazole
c-PTIO: 2-(4-carboxy-2-phenyl)-4,4,5,5-tetramethylimidazoline-1-oxyl-3-oxide
CTK: cytokinin
H_2_O_2_: hydrogen peroxide
IAA: indole-3-acetic acid
JA: jasmonic acid
L-NAME: N-nitro-l-arginine methyl ester
NO: Nitric oxide
NOS: NO synthase
NR: Nitrate reductase
SA: salicylic acid
SNAP: S-nitroso-N-acerylpenicillamine

## References

[1] Grove MD, Spencer GF, Rohwedder WK, Mandava N, Worley JF, Warthen JD, Steffens GL, Flippen-Anderson JL, Cook JC (1979). Brassinolide, a plant growth-promoting steroid isolated from *Brassica napus* pollen. Nature.

[2] Kurepin LV, Bey MA, Back TG, Pharis RP (2016). Structure–function relationships of four stereoisomers of a Brassinolide mimetic on hypocotyl and root elongation of the Brassinosteroid-deficient det2-1 mutant of *Arabidopsis*. J Plant Growth Regul.

[3] Kang Y-Y, Guo S-R, Li J, Duan J-J (2007). Effects of 24-Epibrassinolide on antioxidant system in cucumber seedling roots under hypoxia stress. Agric Sci China.

[4] Aghdam MS, Mohammadkhani N (2014). Enhancement of chilling stress tolerance of tomato fruit by postharvest Brassinolide treatment. Food Bioprocess Technol.

[5] Tanveer M, Shahzad B, Sharma A, Biju S, Bhardwaj R (2018). 24-Epibrassinolide; an active brassinolide and its role in salt stress tolerance in plants: a review. Plant Physiol Biochem.

[6] Sharma P, Kumar A, Bhardwaj R (2016). Plant steroidal hormone epibrassinolide regulate – heavy metal stress tolerance in *Oryza sativa* L. by modulating antioxidant defense expression. Environ Exp Bot.

[7] Anjum SA, Wang LC, Farooq M, Hussain M, Xue LL, Zou CM (2011). Brassinolide application improves the drought tolerance in maize through modulation of enzymatic antioxidants and Leaf gas exchange. J Agron Crop Sci.

[8] Yuan L, Zhu S, Shu S, Sun J, Guo S (2015). Regulation of 2,4-epibrassinolide on mineral nutrient uptake and ion distribution in Ca(NO _3_ ) _2_ stressed cucumber plants ☆. J Plant Physiol.

[9] Zhao G, Xu H, Zhang P, Su X, Zhao H (2017). Effects of 2,4-epibrassinolide on photosynthesis and Rubisco activase gene expression in *Triticum aestivum* L. seedlings under a combination of drought and heat stress. Plant Growth Regul.

[10] Lu Z, Huang M, Ge D-P, Yang Y-H, Cai X-N, Qin P, She J-M (2003). Effect of brassinolide on callus growth and regeneration in *Spartina patens* (Poaceae). Plant Cell Tissue Organ Cult.

[11] Shrestha S, Sturgeon A, Shashkov P, Shatrov A (2007). The effect of different concentration of Epibrassinolide on chlorophyll, protein and anthocyanin content and peroxidase activity in excised red cabbage ( *Brassica Oleraceae* L.) cotyledons. Biotechnol Biotechnol Equip.

[12] Asami T, Min YK, Nagata N, Yamagishi K, Takatsuto S, Fujioka S, Murofushi N, Yamaguchi I, Yoshida S (2000). Characterization of brassinazole, a triazole-type brassinosteroid biosynthesis inhibitor. Plant Physiol.

[13] Sanz L, Albertos P, Mateos I, Sánchezvicente I, Lechón T, Fernándezmarcos M, Lorenzo O (2015). Nitric oxide (NO) and phytohormones crosstalk during early plant development. J Exp Bot.

[14] Gouvêa CMCP, Souza JF, Magalhães ACN, Martins IS (1997). NO·–releasing substances that induce growth elongation in maize root segments. Plant Growth Regul.

[15] Neill SJ, Desikan R, Hancock JT (2003). Nitric oxide signaling in plants. New Phytol New Phytologist.

[16] Parani M, Rudrabhatla S, Myers R, Weirich H, Smith B, Leaman DW, Goldman SL (2004). Microarray analysis of nitric oxide responsive transcripts in *Arabidopsis*. Plant Biotechnol J.

[17] Delledonne M (2005). NO news is good news for plants. Curr Opin Plant Biol.

[18] Besson-Bard A, Pugin A, Wendehenne D (2008). New insights into nitric oxide signaling in plants. Annu Rev Plant Biol.

[19] Hancock JT, Wilson HR, Neill SJ (2017). Nitric oxide Signalling in plants: John Wiley & Sons, ltd.

[20] Correa-Aragunde N, Foresi N, Lamattina L (2015). Nitric oxide is a ubiquitous signal for maintaining redox balance in plant cells: regulation of ascorbate peroxidase as a case study. J Exp Bot.

[21] Astier J, Jeandroz S, Wendehenne D (2018). Nitric oxide synthase in plants: the surprise from algae. Plant Sci.

[22] Zhu Y, Liao W, Wang M, Niu L, Xu Q, Jin X (2016). Nitric oxide is required for hydrogen gas-induced adventitious root formation in cucumber. J Plant Physiol.

[23] Tossi V, Lamattina L, Cassia R (2013). Pharmacological and genetical evidence supporting nitric oxide requirement for 2,4-epibrassinolide regulation of root architecture in *Arabidopsis thaliana*. Plant Signal Behav.

[24] Zottini M, Costa A, De MR, Ruzzene M, Carimi F, Lo SF (2007). Salicylic acid activates nitric oxide synthesis in *Arabidopsis*. J Exp Bot.

[25] Tun NN, Livaja M, Kieber JJ, Scherer GF (2008). Zeatin-induced nitric oxide (NO) biosynthesis in *Arabidopsis thaliana* mutants of NO biosynthesis and of two-component signaling genes. New Phytol.

[26] Huang X, Stettmaier K, Michel C, Hutzler P, Mueller MJ, Durner J (2004). Nitric oxide is induced by wounding and influences jasmonic acid signaling in *Arabidopsis thaliana*. Planta.

[27] Pagnussat GC, Simontacchi M, Puntarulo S, Lamattina L (2002). Nitric oxide is required for root organogenesis. Plant Physiol.

[28] Xiaoting XU, Xin J, Liao W, University GA: Nitric Oxide is Involved in Ethylene-induced Adventitious Root Development in Cucumber Explants. *Acta Horticulturae Sinica* 2017.

[29] Pagnussat GC, Lanteri ML, Lamattina L (2003). Nitric oxide and cyclic GMP are messengers in the indole acetic acid-induced adventitious rooting process. Plant Physiol.

[30] Liao W, Huang G, Yu J, Zhang M, Shi X (2011). Nitric oxide and hydrogen peroxide are involved in indole-3-butyric acid-induced adventitious root development in marigold. J Hortic Sci Biotechnol.

[31] Pacurar DI, Perrone I, Bellini C (2014). Auxin is a central player in the hormone cross-talks that control adventitious rooting. Physiol Plant.

[32] Druege U, Hilo A, Perez-Perez JM, Klopotek Y, Acosta M, Shahinnia F, Zerche S, Franken P, Hajirezaei MR (2019). Molecular and physiological control of adventitious rooting in cuttings: phytohormone action meets resource allocation. Ann Bot.

[33] Zhang A, Zhang J, Zhang J, Ye N, Zhang H, Tan M, Jiang M (2011). Nitric oxide mediates brassinosteroid-induced ABA biosynthesis involved in oxidative stress tolerance in maize leaves. Plant Cell Physiol.

[34] Deng XG, Zhu T, Zou LJ, Han XY, Zhou X, Xi DH, Zhang DW, Lin HH (2016). Orchestration of hydrogen peroxide and nitric oxide in brassinosteroid-mediated systemic virus resistance in *Nicotiana benthamiana*. Plant Journal for Cell & Molecular Biology.

[35] Kwak MS, Kim IH, Kim SK, Han TJ (2009). Effects of Brassinolide with naphthalene acetic acid on the formation of adventitious roots, Trichome-like roots and Calli from cultured tobacco leaf segments, and the expression patterns of CNT103. Journal of Plant Biology.

[36] Karpets YV, Kolupaev YE (2018). Participation of nitric oxide in 24-Epibrassinolide-induced heat resistance of wheat coleoptiles: functional interactions of nitric oxide with reactive oxygen species and Ca ions. Russ J Plant Physiol.

[37] Livak KJ, Schmittgen TD (2001). Analysis of relative gene expression data using real-time quantitative PCR and the 2^−ΔΔCT^ method. Methods.

[38] Müssig C, Shin GH, Altmann T (2003). Brassinosteroids promote root growth in Arabidopsis. Plant Physiol.

[39] Guan M, Roddick JG (1988). Epibrassinolide-inhibition of development of excised, adventitious and intact roots of tomato (*Lycopersicon esculentum*): comparison with the effects of steroidal estrogens. Physiol Plant.

[40] Guan M, Roddick JG (2010). Comparison of the effects of epibrassinolide and steroidal estrogens on adventitious root growth and early shoot development in mung bean cuttings. Physiol Plant.

[41] Nagata N, Min YK, Nakano T, Asami T, Yoshida S (2000). Treatment of dark-grown *Arabidopsis thaliana* with a brassinosteroid-biosynthesis inhibitor, brassinazole, induces some characteristics of light-grown plants. Planta.

[42] Ali B, Hayat S, Ahmad A (2007). 28-Homobrassinolide ameliorates the saline stress in chickpea (*Cicer arietinum* L.). Environ Exp Bot.

[43] Ali B, Hayat S, Fariduddin Q, Ahmad A (2008). 24-Epibrassinolide protects against the stress generated by salinity and nickel in *Brassica juncea*. Chemosphere.

[44] Fariduddin Q, Khanam S, Hasan SA, Ali B, Hayat S, Ahmad A (2009). Effect of 28-homobrassinolide on the drought stress-induced changes in photosynthesis and antioxidant system of *Brassica juncea* L. Acta Physiol Plant.

[45] Engin H, Gokbayrak Z (2016). Effects of 22S, 23S-homobrassinolide and gibberellic acid on formation of double fruits in ‘Bing’ sweet cherry. Acta Hortic.

[46] Yuan LB, Peng ZH, Zhi TT, Zho Z, Liu Y, Zhu Q, Xiong XY, Ren CM (2015). Brassinosteroid enhances cytokinin-induced anthocyanin biosynthesis in *Arabidopsis* seedlings. Biol Plant.

[47] Sasse JM (2010). Brassinolide-induced elongation and auxin. Physiol Plant.

[48] Nakamura A (2003). Brassinolide induces IAA5, IAA19, and DR5, a synthetic Auxin response element in *Arabidopsis*, implying a cross talk point of Brassinosteroid and Auxin signaling. Plant Physiol.

[49] Vandenbussche F, Callebert P, Zadnikova P, Benkova E, Van Der Straeten D (2013). Brassinosteroid control of shoot gravitropism interacts with ethylene and depends on auxin signaling components. Am J Bot.

[50] He Y, Zhang H, Sun Z, Li J, Hong G, Zhu Q, Zhou X, Macfarlane S, Yan F, Chen J (2017). Jasmonic acid-mediated defense suppresses brassinosteroid-mediated susceptibility to Rice black streaked dwarf virus infection in rice. New Phytol.

[51] Li X-P, Xu Q-Q, Liao W-B, Ma Z-J, Xu X-T, Wang M, Ren P-J, Niu L-J, Jin X, Zhu Y-C (2016). Hydrogen peroxide is involved in abscisic acid-induced adventitious rooting in cucumber (*Cucumis sativus* L.) under drought stress. Journal of Plant Biology.

[52] Chen Y, Wang M, Hu L, Liao W, Dawuda MM, Li C (2017). Carbon Monoxide Is Involved in Hydrogen Gas-Induced Adventitious Root Development in Cucumber under Simulated Drought Stress. Front Plant Sci.

[53] Liao W, Xiao H, Zhang M (2009). Role and relationship of nitric oxide and hydrogen peroxide in adventitious root development of marigold. Acta Physiol Plant.

[54] Xu X-T, Jin X, Liao W-B, Dawuda MM, Li X-P, Wang M, Niu L-J, Ren P-J, Zhu Y-C (2017). Nitric oxide is involved in ethylene-induced adventitious root development in cucumber (*Cucumis sativus* L.) explants. Sci Hortic.

[55] Jangid KK, Dwivedi P (2017). Physiological and biochemical changes by nitric oxide and brassinosteroid in tomato (*Lycopersicon esculentum* Mill.) under drought stress. Acta Physiol Plant.

[56] Li X, Zhang L, Ahammed GJ, Li ZX, Wei JP, Shen C, Yan P, Zhang LP, Han WY (2017). Nitric oxide mediates brassinosteroid-induced flavonoid biosynthesis in *Camellia sinensis* L. J Plant Physiol.

[57] Qi F, Xiang Z, Kou N, Cui W, Xu D, Wang R, Zhu D, Shen W (2017). Nitric oxide is involved in methane-induced adventitious root formation in cucumber. Physiol Plant.

[58] Jin X, Liao DW, Yu JH, Ren MP, Dawuda MM, Wang MM, Niu ML, Li MX, Xu MX: Nitric Oxide Is Involved in Ethylene-Induced Adventitious Rooting in M. Canadian Journal of Plant Science 2017.

[59] Xuan W, Xu S, Li M, Han B, Zhang B, Zhang J, Lin Y, Huang J, Shen W, Cui J (2012). Nitric oxide is involved in hemin-induced cucumber adventitious rooting process. J Plant Physiol.

[60] Chen T, Yuan F, Song J, Wang B (2016). Nitric oxide participates in waterlogging tolerance through enhanced adventitious root formation in the euhalophyte *Suaeda salsa*. Funct Plant Biol.

[61] Desikan R, Griffiths R, Hancock J, Neill S (2002). A new role for an old enzyme: nitrate Reductase-mediated nitric oxide generation is required for Abscisic acid-induced Stomatal closure in *Arabidopsis thaliana*. Proc Natl Acad Sci U S A.

[62] Lambeck I, Chi J-C, Krizowski S, Mueller S, Mehlmer N, Teige M, Fischer K, Schwarz G (2010). Kinetic analysis of 14-3-3-inhibited *Arabidopsis thaliana* nitrate Reductase. Biochemistry.

[63] Athwal GS, Huber JL, Huber SC (1998). Phosphorylated nitrate Reductase and 14-3-3 proteins. Plant Physiol.

